# Supplementary material for: Are carbon emissions trading and green financial instruments synergistic? -Comprehensive quantitative research based on content analysis
Source: PLoS One. 2024 Mar 7;19(3):e0298601. doi: 10.1371/journal.pone.0298601 (PMC10919601; doi:10.1371/journal.pone.0298601)
Supplement: S1 Data — (ZIP) [file pone.0298601.s001.zip › Data/地级市Z_F工作报告.zip!/╜¡╦╒/│ú╓▌╩╨/└┤╫╘╣1⁄2╓┌║┼í╛┬φ┐╦╩2╛▌═°í┐_2014.docx]

各位代表：

　　现在，我代表常州市人民政府向大会作工作报告，请予审议，并请市政协各位委员和其他列席人员提出意见。

　　一、2013年工作回顾

　　过去的一年，面对严峻复杂的国内外宏观经济形势，市政府在市委的正确领导和市人大、市政协的监督支持下，团结依靠全市人民，紧扣科学发展主题和转变经济发展方式主线，坚持稳中求进的工作总基调和又好又快的工作导向，积极抢抓苏南现代化示范区建设的机遇，扎实做好稳增长、调结构、抓创新、惠民生、促和谐等各项工作，经济社会保持了持续稳定发展的良好势头，较好地完成了年初确定的各项目标任务。

　　(一)促进经济稳定增长，综合实力不断增强。预计全市实现地区生产总值4350亿元，比上年增长10.9%。公共财政预算收入达408.9亿元，增长7.9%。完成固定资产投资2900亿元，增长18.2%，其中工业投入1540亿元，增长16.6%。规模以上工业总产值首次跨上万亿元台阶，达10070亿元，增长11.5%。实现服务业增加值1975亿元，占地区生产总值的比重达45.4%。完成社会消费品零售总额1587亿元，增长13%以上。注册外资实际到账35亿美元，增长5%，新引进14个协议外资超亿美元项目。完成进出口总额290.5亿美元。主要农产品生产实现平稳增长，农业现代化建设加快推进。大力发展县域经济，金坛、溧阳经济实力得到新提升。

　　(二)加快转型创新步伐，发展质态不断提升。出台了培育发展战略性新兴产业、现代服务业和金融业三年行动计划，制定了推进十大产业链建设、传统优势产业转型升级“双百”行动计划和创新型企业“十百千”计划等推进工业经济转型升级的文件，采取有效措施，不断促进产业结构优化升级，新兴产业产值增长26%。积极推进重大项目建设，每月召开重点项目推进会，大力招引重大项目，重点项目总体进展良好，完成年度投资计划105.2%。龙头企业支撑作用进一步增强，中天钢铁成为首家营业收入超千亿企业，超百亿工业企业达14家。科技创新成效显著，研发经费支出占地区生产总值比重达2.6%，高新技术产值占规上工业产值比重达41.3%，万人发明专利拥有量超10件。高新技术企业超860家，孵化器面积超600万平方米，在孵企业超4900家。国家创新型科技园区预计实现营业总收入超1285亿元，成立江苏中科院智能科学技术应用研究院，实现了省属公共研发平台的重大突破。首次设立“市长质量奖”。实施两批“龙城英才计划”，签约引进领军型创新创业人才项目478个，其中国家“千人计划”特聘专家155名。金融业规模不断扩大，全市金融机构本外币存贷款余额分别增长10%、11%以上。新设平安银行常州分行、南京银行常州分行、苏州银行常州分行和中成村镇银行4家金融机构，全市银行业金融机构达25家。旅游业快速发展，接待国内外游客人数、旅游总收入分别增长16%和20%，天目湖旅游度假区成为我市第二家国家5A级旅游景区。文化产业稳步增长，文化产业增加值占地区生产总值比重突破5%。

　　(三)大力建设美丽常州，城乡环境不断优化。坚持新城建设与旧城改造统筹推进，钟楼新城、凤凰新城等一批城市新片区项目加快实施，背街小巷面貌得到改善，“三改”工作有序展开，改造户数2869户、改造面积57万平方米。加快基础设施建设步伐，龙城大道地道、晋陵南路等城市骨干道路建成通车，锡溧漕河、丹金溧漕河金坛段建成通航，常溧高速公路、青洋路高架北延、玉龙路、河海东路等重点工程顺利推进，新沟河等一批水利重点工程启动建设，常州机场改扩建工程全面完成，机场综合保障能力实现新跃升。城乡绿化建设取得新成效，丁塘河湿地公园、新龙生态林等一批公园绿地加快建设，全市城镇绿化覆盖率达37.3%。成功举办第八届中国花博会，入园参观游客达268万人次。城市管理水平得到提升，市容面貌进一步改善。城乡一体化建设加快推进，启动实施100个中心镇重点项目，全面完成全市村庄环境整治任务。生态文明建设扎实推进，太湖治水和市区河道水环境整治取得新进展，“禁燃区”面积扩大到230平方公里，关闭100家化工、印染企业，我市连续两年在全省生态文明建设工程综合考核中名列第二。荣获国家生态市、省人居环境城市称号。

　　(四)繁荣发展社会事业，人民生活不断改善。着力办好民生实事，十件为民办实事项目全面完成。积极推进居民增收，预计城乡居民收入分别增长10%、11%以上。就业工作扎实推进，城镇新增就业12万人，城镇登记失业率控制在4%以内。社会保障水平不断提高，企业职工养老、职工医疗、失业保险三大保险综合覆盖率保持在98%以上，连续9年上调企业退休人员基本养老金。以辖市区为单位，实现城乡最低生活保障水平一体化。新农合住院费用实际补偿比例超过53%。新开工各类保障房22668套(户)，成功创建省住房保障体系建设示范市。新增机构养老床位1650张，居家养老服务中心(站)实现街道(镇)和城市社区全覆盖。教育现代化扎实推进，全省高考文理科第一名花落常州，两市五区高标准通过全国义务教育基本均衡县(市、区)督导评估，开工建设市五中等30个教育项目，常州技师学院新校区建成投用。国家历史文化名城建设取得新成效，青果巷、前后北岸历史文化街区保护利用工程有序实施，大运河常州段“申遗”通过联合国专家现场验收，舞蹈《香脆萝卜干》、小品《救》荣获全国群众文化最高奖“群星奖”。医疗机构布局进一步优化，群众就医条件不断改善，钟楼三级医院建设项目顺利开工。体育事业蓬勃发展，全市城市(城关镇)社区全部建成“10分钟体育健身圈”，常州运动员在第十二届全运会上获得10枚金牌，创历史最好成绩。稳步推进智慧城市各领域重大项目建设，15个wifi免费试点区域建成开通，完成100个智慧社区建设任务，数字常州地理空间框架基本建成。平安常州建设取得新进展，荣获全国社会治安综合治理最高奖“长安杯”。与此同时，粮食供销、安全生产、人武民防、双拥工作、外事侨务、对台事务、审计统计、海关国检、质监药监、工商物价、司法行政、海事边防、邮政供电、档案方志、防震减灾、应急管理、民族宗教、妇女儿童、人口计生、残疾人事业、老龄事业和关心下一代等工作都取得了新成绩。

　　(五)加强政府自身建设，行政效能不断提高。积极转变政府职能，不断创新行政管理方式，增强政府公信力和执行力。加快行政审批制度改革步伐，取消和下放行政审批事项85项，对重点建设项目实施审批代办制。全面增强“三合一”阳光政务平台服务功能，开通网上远程审批服务通道，对审批服务事项实施网上公示。加强机关作风建设，认真贯彻落实中央、省和我市关于改进工作作风、密切联系群众的各项规定，大力倡导求真务实、狠抓落实的工作作风。着力推进依法行政，自觉接受市人大、市政协及各民主党派的监督，办理人大代表建议、政协提案435件，妥善解决了一批经济社会发展方面的问题。

　　各位代表!去年各项成绩的取得，是全市上下在省委、省政府和市委的正确领导下，团结拼搏、攻坚克难、开拓创新的结果。在此，我代表市人民政府，向辛勤工作在各个领域的全市人民，向市人大代表、政协委员，向市各民主党派、工商联、各人民团体、各界人士和离退休老同志，向驻常人民解放军、武警官兵、公安政法干警和部省驻常单位，向所有关心、支持、参与常州建设和发展的中外投资者、科技工作者、务工人员和海内外朋友们，致以衷心的感谢和崇高的敬意!

　　在肯定成绩的同时，我们也清醒地认识到面临的挑战和工作中存在的问题，主要表现在：部分企业生产经营面临前所未有的困难，公共财政预算收入等指标增速较低，城乡居民收入增长趋缓;产业结构依然偏重，重大项目不多，转型发展面临较大压力;大气环境质量有待进一步改善，节能减排任务十分艰巨;政府服务效能还需要进一步提升，机关作风建设有待进一步加强。对此，我们一定会高度重视，采取有针对性的措施，下大力气加以解决。

　　二、2014年工作安排

　　2014年是全面深化改革的第一年，也是加快常州现代化进程的关键之年。今年政府工作的总体要求是：深入贯彻落实党的十八大和十八届三中全会精神，按照市委十一届六次全会部署，紧紧围绕苏南现代化示范区建设目标，坚持稳中求进的工作总基调，把改革创新贯穿于经济社会发展的各个领域各个环节，以“重大项目突破年”活动为抓手，加快转型升级、狠抓项目建设、注重产城融合、推动生态文明、统筹城乡发展、着力改善民生，不断提高经济发展质量和效益，实现经济持续健康发展和社会和谐稳定。 今年全市经济社会发展的主要预期目标是：地区生产总值增长10%左右;公共财政预算收入增长8%左右;固定资产投资增长17%;社会消费品零售总额增长13%;外贸进出口总额增长5%左右;注册外资实际到账保持一定增长;城乡居民收入增长10%以上;居民消费价格总水平涨幅控制在3.5%以内;城镇登记失业率控制在4%以内;全面完成省下达的节能减排任务。

　　为实现上述目标，我们将重点做好八个方面的工作：

　　(一)着力推动经济持续健康发展，提高城市综合实力1.推进重大项目。强力推进“重大项目突破年”活动，加大项目招引力度，采用小分队招商、精准招商、专业招商等多种方式，重点引进新兴产业高端项目以及产业链、价值链高端环节和缺失环节，力争在重要产业链的重大项目建设上取得突破。进一步加大投入力度，优化投入结构，166个市级以上重点项目完成年度投资703亿元。完善重大项目推进机制，创新项目组织方式，加大项目考核力度，坚持每月召开重点项目推进会，及时协调解决项目推进中的难题，确保项目引得进、落得下、推得动。

　　2.壮大实体经济。进一步提振全社会发展实体经济的信心，不断优化经济发展环境。大力发展民营经济，全年新增私营企业1.2万户、个体工商户3万户。做大做强龙头企业，鼓励龙头企业通过技术改造、品牌建设、质量提升、兼并重组等多种方式壮大企业实力。加大金融支持实体经济力度，力争银行业金融机构各项贷款增速高于我市地区生产总值增幅与物价指数增幅之和。大力推动企业上市，力争有5家企业在境内外上市，后备上市企业达100家。

　　3.提升区域经济。深入实施促进县域经济发展的各项政策，进一步推动产业、科技、项目和基础设施“四个西进”，深化武进与金坛、新北与溧阳挂钩合作，加大对县域经济发展的支持力度。金坛、溧阳要加快重大项目、开发园区建设步伐，积极发展特色优势产业，努力提升县域经济发展的质量和水平。武进、新北要重点围绕十大产业链建设，大力建设创新创业平台，突破引进一批重大项目，进一步提升对全市经济的贡献份额。天宁、钟楼和戚墅堰区要大力发展现代服务业和都市工业，增强经济持续发展能力。

　　(二)全力推进转型升级，提升发展质量和效益1.加快工业经济转型升级。大力推进十大产业链建设、传统优势产业转型升级“双百”行动计划和创新型企业培育“十百千”计划。采取优化发展布局、鼓励资本运作、加大政策支持等措施，不断提升工业经济发展质态，努力形成十大产业链建设、传统优势产业转型升级、创新型企业培育“三位一体”的工业经济转型升级新路。力争十大产业链产值占规模以上工业比重提高2个百分点，五个传统优势产业规模以上企业实现产值9300亿元，实现利税680亿元，百项重点技改项目完成投资100亿元以上。

　　2.加快现代服务业发展。在巩固提升传统商贸服务业发展水平的基础上，加快现代服务业发展步伐，突破发展生产性服务业、提升发展消费性服务业、培育发展民生性服务业，推进服务业结构优化、水平提升，确保服务业增加值占地区生产总值的比重提高1.5个百分点以上。推动金融服务业发展，积极推进东经120金融商务区建设，加快把我市建成区域性的资本运作中心、资产管理中心和金融服务中心。加快旅游业发展，推进东方盐湖城一期等旅游业重点项目建设，进一步丰富乡村旅游、文化旅游等旅游产品，旅游业总收入增长17%。加快电子商务发展，大力培育壮大电子商务骨干企业，推动电子商务企业集聚发展，积极推进制造业、商贸流通业与电子商务融合发展。

　　3.加快农业现代化进程。大力推进现代农业产业园区建设，不断提升农业组织化、规模化、科技化和产业化水平。优化农业结构，大力发展生态农业、休闲观光农业、设施农业，提升产出效益，增强生态功能。继续加强农业基础建设，新增高标准农田8万亩。推进“亩产吨粮市”创建活动，新增市级农业龙头企业20家以上，建设市级以上农业产业园区10个，现代化粮食仓容占比达到50%。

　　(三)加快实施创新驱动，建设创新型城市

　　1.培育创新企业。鼓励企业加大研发投入，研发经费支出占地区生产总值比重达2.8%左右。突出企业创新主体地位，加快培育创新型企业。打造一批创新型领军企业，支持和推动龙头骨干企业加大研发投入、建立研发机构，承担国家和省重大科技成果产业化项目和产学研合作项目。培育一批科技型上市企业，加大对科技型中小企业上市的培育和支持力度。壮大高新技术企业，累计突破900家。

　　2.建设创新载体。推进科技园区建设，加快构建“一核两区三园多基地”的区域创新布局，常州科教城加快建设成为国际一流的高职教育和国际化产学研协同创新基地，高起点规划建设江苏中科院智能科学技术应用研究院，常州高新区、武进高新区要迈入全国高新区先进行列，西太湖科技产业园、江苏中关村科技产业园、华罗庚科技产业园要打造成为具有区域特色的创新高地。完善现有孵化器的功能定位，引导社会资本和骨干企业建设孵化器，新增孵化面积100万平方米，在孵企业超过5500家。推进创新平台建设，重点建设1-2家产业共性技术研发平台，积极发展20家左右科技企业孵化平台，支持发展10家左右产业技术公共服务平台。

　　3.集聚创新创业人才。大力实施“龙城英才计划”，各级政府要引进拥有核心知识产权、有成功创业经历的创业型人才，提升我市产业发展水平。鼓励企业大力引进掌握前沿技术、创新能力强的创新型人才，加大高技能人才队伍建设力度，每万名劳动者中高技能人才数达600名。进一步优化人才创新创业环境，完善创新创业人才政策，健全人才引进、培养、使用、激励保障等工作机制，充分调动各类人才的创新创造活力。

　　(四)全面推进改革开放，激发科学发展新活力1.推进重点领域改革。深化科技体制改革，完善产学研协同创新机制，积极推进科技与金融、人才与资本有效结合，释放科技创新活力。推进文化体制改革，创新文化产业多元投入机制，鼓励非公有制文化企业发展，完善市级国有文化企业管理机制，激发文化企业内生动力。推进医药卫生体制改革，加快县级公立医院综合改革进程，建立稳定、长效的县级公立医院补偿新机制。继续深化农村“三大合作”改革，抓好土地承包经营权、宅基地使用权、集体建设用地使用权的确权登记颁证，加快建立农村产权交易中心、土地承包经营权流转市场和城乡统一的建设用地市场，探索建立农民承包地经营权、宅基地使用权和集体建设用地抵押、担保机制。

　　2.提升外经贸工作水平。加强与上海自贸区对接，充分利用上海自贸区的“溢出效应”。优化外资结构，紧盯世界500强和行业龙头企业，强化产业链招商，大力引进跨国公司地区总部和研发、采购、销售、物流等功能性机构，全力推动一批重大高端产业项目落户。推进外贸转型，坚持稳出口与促进口并重，鼓励企业加大国际市场开拓力度，加快高新技术产品、机电产品和服务贸易、文化产品出口，支持企业进口国际先进生产设备、关键技术和重要生产资料。加快“走出去”步伐，鼓励有条件的企业到境外并购知名品牌和优质资产，开展技术和产品研发，构建国际营销网络;积极推动境外工程承包，带动设备、技术输出。

　　3.推动开发区转型发展。优化发展规划，鼓励各开发区按照我市产业链发展的要求，结合自身资源条件和产业基础，重点打造一批“特色园区”，形成各具特色的主导产业，提升园区核心竞争力。推进产城融合，促使开发区从形态开发向功能开发转变，支持符合条件的开发区扩区升级，推动金坛、溧阳开发区升格为国家级经济技术开发区，推动常州出口加工区、武进出口加工区升格为综合保税区，推动西太湖科技产业园、江苏中关村科技产业园、华罗庚科技产业园、武进绿色建筑产业集聚示范区、中德创新园区、航空产业园等载体建设;按照园区国际化的要求，推动国际合作园区建设，提高开发区管理水平。坚持集聚集约发展，进一步提高开发区的投入强度和产出水平，努力把开发区建成生态工业示范区和低碳经济示范区，继续引领全市经济社会发展。

　　(五)加大城乡统筹发展力度，建设现代化精品城市1.推进基础设施建设。加快常溧高速公路建设和常宜、溧广高速公路前期工作。全力推进地铁和城市路网疏导工程，开工建设地铁1号线，完成新堂北路翠竹段、红梅南路等地铁疏导路网建设工程，青洋路高架北延、河海东路拓宽等工程建成通车，开工建设兴东路、劳动西路、白云南路等道路。加快县域快速干道建设，金武路快速化改造工程主体建成，延政西路西延工程基本建成，S122、S263等国省干线公路加快建设。积极推进输变电工程和一流配农网建设。实施水利建设工程，完成新沟河工程年度投资目标，加快新孟河工程前期工作，实施长江堤防提升加固工程、丁塘港拓浚工程和澡港河整治工程，完成千亩以上圩堤加固150公里以上。

　　2.提升城市功能。围绕现代化城市建设，做好区域协调、空间发展、产业发展等规划编制工作，推动土地规划、空间规划、产业规划、生态规划“四规合一”。加快城市新片区建设进程，着力推进西太湖科技城、新龙国际商务城、凤凰新城、钟楼新城等片区重要功能项目建设，加快东大门建设步伐，尽快形成现代化城区新形象。加快中心城区改造提升步伐，继续实施“三改”工程，推进31个旧城改造项目。提高公用事业保障能力，实施应急水源一期工程和雨污水设施能力提升工程，开工建设戚墅堰污水处理厂三期和天然气利用三期等工程。

　　3.提高城市管理水平。深入开展城市环境综合整治“9313”行动计划，突出解决城市环境薄弱地段脏乱差问题，重点提升高架沿线、城郊结合部、城中村等区域的环境质量。巩固提升城市长效管理水平，推进管理力量向社区、薄弱地段延伸，实现城市管理全覆盖、常态化。加强对工业固体废弃物、建筑垃圾等废弃物的综合利用和管理，完成餐厨废弃物综合处置一期工程，提升环卫保障管理水平。

　　4.推进城乡一体化发展。推动城乡统筹发展，构建城乡一体化发展新格局。加快小城镇建设步伐，推进8个重点中心镇建设，打造一批专业镇、特色镇，提升小城镇的辐射带动能力。加快新农村建设步伐，深入实施强村富民工程，进一步做强村级集体经济，金坛、溧阳行政村(涉农社区)村级年收入达70万元以上，常武地区行政村(涉农社区)村级年收入达100万元。继续改善农村人居环境，城镇污水集中处理率达95%，镇村生活垃圾无害化处理率达100%，建成10个美丽乡村示范村。探索建立生态补偿机制，在水稻田、水源地、生态公益林、生态湿地等重点领域开展生态补偿试点工作。

　　(六)加强生态文明建设，建设美丽常州

　　1.建设生态绿城。围绕建设宜居城市，大力推进以“增核、扩绿、联网”为主要内容的生态绿城建设。实施生态源保护、中心城区绿地、环城郊野公园、生态绿道等生态绿城建设工程，建设横塘河、永安河等一批湿地公园，推进白荡河等公共绿地建设，对小黄山、长江水源地实施生态源保护，在长江路、延政路、南运河等沿线建设生态绿道，建设“怀德桥—桃园滨河绿地一期”城市慢行系统，完成翠竹公园、蔷薇园改造工程，营造良好的城乡绿化环境。做好花博会园区后续开发利用工作。

　　2.推进环境综合整治。深入实施清水蓝天宜居工程，强化环保联动执法，不断巩固提升全市环境质量。实施河道水质再提升工程，深入开展工业污水和农业面源污染治理，不断改善141条已整治河道水质，提升澡港河等7条河道生态功能，太湖水治理完成省定治理任务。大力开展大气环境综合整治，强化大气污染源头控制，深化工业污染治理，加强机动车污染防治，开展扬尘污染治理，进一步改善我市的空气质量。

　　3.狠抓节能减排。组织实施一批节能减排重点工程，对10家热电企业实施脱硫、脱硝、除尘提标改造，国电常州发电公司达到新的排放标准。推进炼钢、炼铁、水泥等行业淘汰落后产能，对重污染化工企业实施关停并转。实施清洁生产，完成50家企业的自愿性清洁生产审核和100家企业的强制性清洁生产审核。实施资源综合利用，推进滨江开发区、钟楼开发区等园区循环化试点工程。

　　(七)注重民生为先，推进社会事业加快发展

　　1.加强就业和社会保障。大力推进创业带动就业，城镇新增就业9万人，常州籍高校毕业生就业率达到95%以上。扩大社会保险覆盖面，统筹推进城乡社会保障体系建设，稳步提高社会保障待遇水平。做好廉租住房和公共租赁住房建设工作，发放住房公积金贷款40亿元。加快完善社会救助制度，落实城乡低保标准自然增长机制，加大困难群众医疗救助力度。关爱残疾人和妇女儿童，为市区1万名持证无业残疾人提供免费体检，为20万名适龄妇女免费开展“两癌”筛查，为城区1.5万名适龄儿童开展口腔检查。建立多元化养老模式，提升社区居家养老服务水平，加大养老机构建设力度，推动医养融合发展，积极发展农村区域性的养老服务中心。

　　2.提升文化软实力。完善公共文化服务体系，启动申报省级公共文化服务体系示范区创建，加快文化广场工程建设，扎实推进“文明社区天天乐”和“精彩龙城—文化惠民月”活动。深入推进国家历史文化名城建设，抓好青果巷、前后北岸历史文化街区等一批重点文化项目，深入开展历史文化名镇、名村的保护利用，做好“非遗”抢救性保护工作。大力发展文化产业，推进文化与相关产业融合发展，壮大一批文化产业示范园区和龙头文化企业，力争文化产业增加值占地区生产总值比重达5.5%以上。

　　3.协调发展各项事业。扎实推进教育现代化建设，实施省义务教育优质均衡改革发展示范区、省职业教育创新发展实验区建设。加大教育投入力度，加快推进中小学布局调整和常州大学、常州工学院新校区、殷村职教园建设。加强公共卫生服务体系建设，提高医疗卫生服务水平，市一院综合病房大楼、市七院门急诊病房大楼建成使用，加快钟楼三级医院建设步伐。完善城乡基层卫生服务体系规划，进一步提升基层卫生服务能力。调整完善计划生育政策，启动实施一方是独生子女的夫妇可生育两个孩子的政策。实施公共体育服务体系三年行动计划，高水平建设“10分钟体育健身圈”，全力备战第十八届省运会，承办好中国羽毛球大师赛、环太湖自行车赛等重要赛事。加快智慧城市建设，推动常州高新区智慧城市产业园建设，推进信息基础设施和信息资源共建共享，实施社区信息化、免费WIFI等一批信息惠民工程，提高社会各领域信息技术应用水平。支持工会、共青团、妇联等人民团体工作，进一步加强外事侨务、对台事务、档案方志、民族宗教、防灾减灾、双拥、残疾人事业和关心下一代等工作。

　　4.推进社会管理创新。加强精神文明建设，深化“道德讲堂”建设，倡导见义勇为，提升市民素质。大力建设和谐社区，城乡和谐社区达标率分别达96%和95%。严格落实社区事务准入制，建立政府行政管理与基层自治组织有效衔接和良性互动机制。进一步推动社会稳定风险评估，加强和改进信访工作，完善维护群众权益的工作机制，努力从源头上化解社会矛盾。深入推进“民生警务”，进一步健全应急管理体系，抓好突发事件风险防范，切实加强生产安全、质量安全等综合监管和司法行政、国家安全工作，不断提升“平安常州”建设水平。深化全民国防教育，加强国防动员和后备力量建设，进一步密切军政军民关系。

　　与此同时，今年我们将继续办好推进就业和社会保障等十件为民办实事项目，让广大市民更多更好地享受常州发展的成果。

　　(八)推动职能转变，建设人民满意的服务型政府1.改进工作作风，提升行政服务水平。全面落实中央、省和我市关于改进工作作风、密切联系群众的各项规定，开展党的群众路线教育实践活动。完善服务基层、服务群众的长效机制，建立机关干部与重点工程、重点项目联系挂钩的工作制度，继续开展走访规模以上工业企业活动，着力解决基层和群众反映强烈的热点、难点问题。牢固树立雷厉风行、一抓到底、干就必成的工作作风，高效推进各项工作。加强行政效能监察，大力整治机关“庸懒散慢”等不良作风，着力提高行政效率和执行力。

　　2.转变政府职能，提升公共管理效能。加快行政审批制度改革步伐，以减少审批事项、创新审批方式为重点，进一步优化审批流程，缩短审批时间，减轻企业负担，推进审批事项网上阳光运行，提高行政效能。本届政府任期内市政府部门行政审批项目减少三分之一以上，保留的一般性审批项目办结时限总体缩短一半以上，所有行政审批项目收费一律按标准下限收取。加快机构改革步伐，进一步整合机构职能，规范机构设置，理顺权责关系，完善体制机制，加快形成精干高效的地方政府组织体系。稳妥推进事业单位分类改革。进一步加强政务服务体系建设，努力形成“一办四中心”的运行模式，更好地架起政府与群众联系的“连心桥”。

　　3.加强自身建设，提升政府公信力。坚持依法行政，自觉接受人大的法律监督、工作监督和政协的民主监督，重视审计监督、司法监督和社会公众监督。加强政府系统廉政建设，严格遵守廉洁从政的各项规定，完善惩治和预防腐败体系，强化对重点领域、关键环节、重要岗位权力运行的监督制约，严肃查处各类违法违纪案件，坚决防止损害群众利益行为。大力弘扬艰苦奋斗、勤俭节约的优良传统，严格财政预算管理，确保行政运行经费零增长、“三公”经费负增长，把有限的资源和财力更多地用到改善民生和服务发展上。

　　各位代表，常州正处于加快现代化进程的关键时期。让我们在中共常州市委的正确领导下，进一步解放思想，开拓奋进，扎实工作，为共同创造我市更加美好的明天、谱写中国梦的常州篇章而努力奋斗!
